# Single-cell Raman insights into microbial strategies for sustainable phosphorus mining and recycling

**DOI:** 10.3389/fmicb.2026.1774897

**Published:** 2026-02-16

**Authors:** Pengcheng Sun, Huihui Pan, Dong Cheng, Yishang Ren, Guangxia Ma, Xiaoyan Jing

**Affiliations:** 1 College of Biological Engineering, Qingdao University of Science and Technology, Qingdao, Shandong, China; 2 Single-Cell Center, CAS Key Laboratory of Biofuels, Shandong Key Laboratory of Energy Genetics and Shandong Energy Institute, Qingdao Institute of BioEnergy and Bioprocess Technology, Chinese Academy of Sciences, Qingdao, Shandong, China

**Keywords:** phosphorus-accumulating organisms (PAOs), phosphorus-solubilizing microbiomes (PSMs), resource recovery, single-cell Raman spectroscopy (SCRS), sustainable phosphorus cycle

## Abstract

Phosphorus (P) management faces a dual crisis of resource depletion and eutrophication, underscoring the need for a sustainable P cycling model. This review systematically elaborates on the microorganism-driven “Mobilization, Retention and Buffering” (MRB) strategy to enable sustainable P cycling. In this framework, phosphorus-solubilizing microorganisms (PSMs) mobilize P, while polyphosphate (poly-P)-accumulating organisms (PAOs) ensure efficient P retention and buffering via poly-P storage. We highlight the unique strengths of single-cell Raman spectroscopy (SCRS), including culture-independent and non-destructive analysis at single-cell resolution, and discuss how it supports *in situ* identification, mechanistic characterization, and mining of functional P-cycling bacteria. Finally, we outline SCRS-enabled opportunities to advance the MRB strategy for efficient P recovery, recycling, and utilization.

## Graphical abstract

**Figure:**
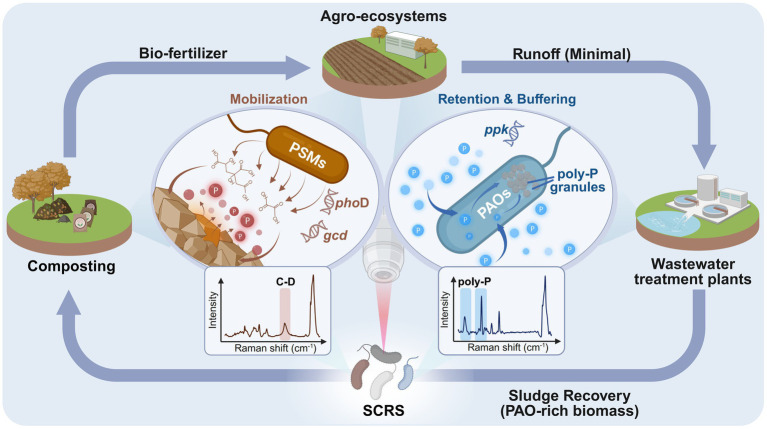

## 1 Introduction

Phosphorus (P) is essential for life and an irreplaceable global strategic resource (Steiner and Geissler, 2018). Modern agriculture relies heavily on phosphate rock, yet economically viable reserves are concentrated in a few countries, with about 70% located in Morocco and Western Sahara (Cordell et al., 2009). In the coming decades, global P demand may outpace supply, threatening food security (Elser and Bennett, 2011; Luo et al., 2024). Meanwhile, phosphate fertilizers in current agriculture exhibit extremely low utilization efficiency, with only ~12.6% being absorbed by plants (Luo et al., 2024). Fertilizers losses through agricultural runoff, coupled with P-rich wastes from municipal, aquaculture, and industrial sources, further drive eutrophication (Goyette et al., 2018; Luo et al., 2024). This coupled challenge of resource scarcity and environmental pollution highlights the need for sustainable, environmentally friendly P utilization models (Solangi et al., 2023).

Microorganisms are key drivers of global elemental cycling (Pang et al., 2024; Nair et al., 2025). In response to fluctuations in P availability across habitats, they have evolved efficient P utilization strategies that support P cycling (Diaz et al., 2022; Peng et al., 2025). In soil, phosphate-solubilizing microorganisms (PSMs) convert insoluble P into bioavailable orthophosphate, promoting crop growth and stress tolerance, thereby performing a “Mobilization” role for P (Pang et al., 2024; Chakraborty et al., 2025; Peng et al., 2025). In parallel, polyphosphate-accumulating organisms (PAOs), which can excessively absorb and store phosphate, intercept free orthophosphate in the soil, reducing nutrient loss caused by runoff and performing a “Retention and Buffering” role for P (Srivastava et al., 2022; Wang et al., 2023b; Ibrahim et al., 2024; Wu et al., 2024). In wastewater biological P removal, the “Retention” of PAOs combined with the “Mobilization” role of PSMs collectively enhances total P removal. The resulting P-rich sludge is further utilized for land, converting P from a pollutant into a recoverable P resource (Diaz et al., 2022; Zhan et al., 2023; He et al., 2025; Kalpakchiev et al., 2025). Theoretically, P cycling driven by this “Mobilization, Retention and Buffering” (MRB) strategy establishes a virtuous closed-loop system, whose core is to increase P supply, reduce losses, and improve recovery to maximize P utilization efficiency. The potential of PSMs and PAOs has been widely recognized, but their application remains limited (Zhou et al., 2025). This is mainly due to the gap between theoretical and actual efficiency and stability of microbe-mediated P cycling, highlighting the need for effective monitoring tools for optimization (Coats et al., 2017; Raymond et al., 2021).

Single-cell Raman spectroscopy (SCRS) is a next-generation physiological method that, based on molecular vibrational scattering, provides chemical and metabolic insights into single cells, including key metabolites like nucleic acids, proteins, lipids, and polysaccharides (Li et al., 2012; Hatzenpichler et al., 2020; Cui et al., 2022). Its non-destructive, label-free nature allows dynamic, quantitative, and in-situ analysis of cell heterogeneity. These advantages are of great significance for analyzing the in-situ mechanisms of PSMs and PAOs (Majed et al., 2009; Petriglieri et al., 2021; Bi et al., 2025). SCRS also offers versatility, integrating with stable isotopes (e.g., ^13^C, ^2^H, ^15^N, ^18^O) to track metabolic fluxes (Berry et al., 2015). Furthermore, its integration with cultivation strategies facilitates the targeted isolation of highly efficient functional strains from in-situ environments, overcoming the limitations of traditional culture methods (Li et al., 2019; Jing et al., 2022).

This review focuses on the MRB strategy for sustainable P utilization. We particularly highlight the unique advantages of SCRS in the *in situ* identifying and mining functional PSMs and PAOs, and its potential in MRB strategies. SCRS offers new perspectives and methods for understanding microbe-mediated P cycling, enhancing the sustainable recovery and utilization of global P resources.

## 2 Application of MRB strategy for sustainable P resource utilization

### 2.1 P cycle and metabolic mechanisms driven by PSMs and PAOs

PSMs are widely distributed in bacteria, fungi, actinomycetes, and cyanobacteria (Rawat et al., 2021). They drive the “Mobilization” stage of the P cycle by converting fixed P into soluble P that can be directly utilized by microorganisms or plants. Inorganic P solubilization is chiefly driven by the pyrroloquinoline quinone-dependent glucose dehydrogenase (PQQ-GDH) encoded by the *gcd* gene, an enzyme that catalyzes the oxidation of sugars (e.g., glucose) to produce large quantities of organic acids. These organic acids not only acidify the rhizosphere microenvironment but also, through potent chelation, break down mineral lattices, thereby releasing immobilized inorganic P (Li G. et al., 2023; Li H. P. et al., 2023; Li Z. et al., 2023; Peng et al., 2025). For organic P mineralization, in response to the chemical heterogeneity of the organic P pool, PSMs have evolved a broad spectrum of extracellular enzyme systems (Sun et al., 2017; Stosiek et al., 2020; Zhang et al., 2025). Notably, these metabolic functions exhibit high transcriptional regulatory plasticity. PSMs can respond not only to P starvation induction via the classic Pho regulatory system but also utilize constitutive enzymes (e.g., PafA) to maintain basal mineralization activity in P-rich habitats. This diverse metabolic mechanism is a key adaptive strategy for coping with environmental P fluctuations (Lidbury et al., 2022) (Table 1).

**Table 1:** Main mechanisms and molecular basis of soil P mobilization by PSMs.

Mechanism category	Specific pathway	Key effectors/enzymes	Substrates/targets	Key genes/gene clusters	References
Inorganic phosphate solubilization	Acidolysis	Organic acids (gluconic, oxalic, citric, lactic acid, etc.)	Insoluble inorganic phosphate (P complexed with Ca^2+^, Fe^3+^, Al^3+^)	*gcd*, *pqq*	Li H. P. et al. (2023); Peng et al. (2025)
	Chelation	Hydroxyl / Carboxyl groups of organic acids	Metal cations (Mg^2+^, Ca^2+^, Fe^3+^)	—	Osmolovskaya et al. (2018)
	Respiratory acidification	CO_2_ (forming carbonic acid/H_2_CO_3_)	Environmental pH	—	Tomar et al. (2024)
Organic phosphorus mineralization	Phosphomonoester hydrolysis (acidic conditions)	Acid phosphatase (ACP)	Phosphomonoesters	*pho*C, *acp*A	Zhang et al. (2025)
	Phosphomonoester hydrolysis (neutral/alkaline conditions)	Alkaline phosphatase (ALP)	Phospholipids, ATP	*pho*D, *pho*A, *pho*X	Wang et al. (2023)
	Phytate degradation	Phytase	Phytate	*app*A, *bpp*	Sun et al. (2017)
	C–P bond cleavage	C–P lyase	Organophosphonates (certain herbicides)	*phn* gene cluste	Stosiek et al. (2020)
Novel/special mechanisms	Constitutive mineralization	Novel phosphatases	Phosphorylated carbohydrates	*paf*A	Lidbury et al. (2022)

PAOs’ retention and buffering capacity fundamentally rely on a reversible aerobic-anaerobic cycle (Table 2). Under aerobic conditions, PAOs oxidize intracellular carbon sources to generate energy, utilizing high-affinity Pst systems and low-affinity Pit systems to uptake phosphate against the concentration gradient (Mino et al., 1998). Concurrently, cells co-transport magnesium (Mg^2+^) and potassium (K^+^) to neutralize negative charges, thereby maintaining charge balance and osmotic stability (Acevedo et al., 2012). Ultimately, intracellular phosphate is converted into poly-P storage under the catalysis of polyphosphate kinase (PPK) (Crocetti et al., 2000). Conversely, under anaerobic conditions, PAOs hydrolyze glycogen and poly-P to energize volatile fatty acid (VFA) assimilation while releasing phosphate (Mino et al., 1998) (Table 2). Through this cycle of anaerobic release and aerobic uptake, PAOs effectively regulate environmental P levels. They occupy distinct ecological niches based on their carbon substrates and electron acceptors. For instance, *Ca. accumulibacter* utilize O_2_ as an electron acceptor; while denitrifying PAOs (DPAOs) like *Ca. dechloromonas* utilize nitrate in an anoxic environment (Goel et al., 2005). Fermentative PAOs, such as *Tetrasphaera,* can ferment complex macromolecules like glucose and amino acids, gaining them a competitive advantage (Close et al., 2021). Recent studies also show that some *Ca. accumulibacter* strains can use light energy for P uptake under O_2_, NO_3_^−^, and NO_2_^−^ deficient conditions (Carvalho et al., 2024). These findings highlight the metabolic diversity and adaptive flexibility of PAOs, underscoring the necessity for further research into their niche-specific mechanisms.

**Table 2:** Metabolic characteristics and energy strategies of different ecotypes of PAOs.

Ecotypes	Representative genus	Anaerobic substrate	Intracellular storage compounds	Electron acceptor	References
Aerobic PAO	*Ca. accumulibacter*	Volatile fatty acids (VFAs)	Polyhydroxyalkanoates (PHA), glycogen	O_2_	Crocetti et al. (2000)
DPAO	*Ca. dechloromonas*	VFA	PHA, glycogen	NO_3_^−^/NO_2_^−^	Goel et al. (2005)
Fermentative PAO	*Tetrasphaera*	Glucose, amino acids	Glycogen	O_2_/NO_3_^−^/NO_2_^−^	Close et al. (2021)
Photoheterotrophic PAO	*Ca. accumulibacter* clades	VFAs/organics	PHA	Light	Carvalho et al. (2024)

### 2.2 Application of MRB strategy for promoting sustainable P recycling

The MRB strategy has proven effective for sustainable P utilization at multiple scales. In agricultural, co-inoculating PSMs and PAOs reduces P leaching by 22.6% and increase the soil’s available P pool by 18.3% (Li et al., 2023c). PSMs enhance wheat P utilization by 91% through hydrolyzing poly-P-rich fertilizers (Khourchi et al., 2023). In composting systems, microbial biomass P increased by 83% with PSM and PAO inoculation, converting organic waste into high-quality bio-slow-release fertilizers (Zhan et al., 2023). PAO-rich biosolids from EBPR treatment of agricultural wastewater aid in recovering high-purity P through technologies like struvite crystallization (Kalpakchiev et al., 2025). Notably, using EBPR activated sludge as P fertilizer improves corn growth, with ~30% of PAOs surviving in the soil, forming a mutualistic network with plant growth-promoting rhizobacteria for sustainable P supply (He et al., 2025). Furthermore, a positive correlation has been found between PAO abundance and plant growth-promoting traits, stress resistance, and alleviation of salt stress (Srivastava et al., 2022). The MRB strategy holds significant potential for P pollution control, legacy soil P activation, and increased crop uptake and yield.

### 2.3 Bottlenecks of traditional detection methods in MRB strategies

Despite the great potential of MRB strategies in agricultural P fixation, transformation, and wastewater P removal, limitations in reaction rate and stability remain (Dou et al., 2025). Traditional methods are hindered by a lagging understanding of core functional groups in complex habitats. These methods typically follow the “culture-first screen-second” model, which involves isolating and screening the PSMs or PAOs under laboratory, followed by screening strains with desirable phenotypes for validation (De Zutter et al., 2022; Fu et al., 2024). However, such strains often face genotype–phenotype mismatches, with poor colonization and unstable performance in environments (Coats et al., 2017; Raymond et al., 2021; Copeland et al., 2025). Therefore, there is an urgent need for new methods to screen strains with natural competitive advantages (Liu et al., 2024). Moreover, the presence of functional genes (e.g., *pqq*C) does not guarantee *in situ* metabolic activity, making it difficult to identify functional executors (Dai et al., 2020). In wastewater P removal, the unculturable nature of key PAO populations, such as *Ca. accumulibacter*, hinders progress and forces reliance on substitute strains that do not represent the real ecological niche, leading to misjudgments (Burow et al., 2008; Yan et al., 2024). More critically, existing mainstream methods are still limited to characterization at the population-average level. This low-resolution perspective not only masks metabolic heterogeneity and interspecies competition at the single-cell level, but also makes it difficult to precisely quantify the dynamic transformations of key intracellular polymers (e.g., poly-P) (Li G. et al., 2023). Overcoming this bottleneck lies in introducing *in situ* characterization techniques with single-cell resolution to unlock the metabolic “black box” of complex microbial communities.

## 3 Advances in the application of SCRS in PSMs and PAOs microorganisms

### 3.1 SCRS enables precise phenotypic identification of in situ PSMs and PAOs

SCRS is a non-invasive, label-free technique that analyzes the internal chemical composition of single cells by capturing molecular vibrations (Wang et al., 2017). In the 400–1800 cm^−1^ wavenumber range, cells produce characteristic spectra known as the “fingerprint region” (Pan et al., 2025). These peaks reflect the metabolic and functional status of cells; allowing for the identification of cellular phenotypes (Li et al., 2012; Hatzenpichler et al., 2020; Cui et al., 2022).

PAOs can uptake P luxuriously, storing it as poly-P intracellularly (Ying et al., 2024). The accumulation of intracellular poly-P is a key feature of PAOs function (Bi et al., 2025). Majed et al. (2009) pioneered the use of Raman spectroscopy for *in situ* identification of intracellular poly-P at the single-cell level. Poly-P characteristic peaks are found at 690–700 cm^−1^ (P–O–P bond stretching) and 1,168–1,177 cm^−1^ (P–O–P bond stretching). The intensity of the latter is linearly correlated with poly-P concentration, serving as a reliable semi-quantitative indicator of PAOs’ P storage capacity (Majed et al., 2009). Furthermore, combining SCRS with fluorescence *in situ* hybridization (FISH) links phylogenetic identity to metabolic function, enhancing the identification of functional microorganisms *in situ*, which is critical for optimizing system stability in mixed-culture processes (Fernando et al., 2019; Petriglieri et al., 2022).

In contrast, PSMs convert insoluble P into bioavailable P extracellularly. Tracking P (^31^P) is challenging due to its single isotope, which prevents stable isotope probing (SIP) (Hatzakis et al., 2008). Recently, SCRS combined with deuterium (D) isotope probing (Raman-DIP) has enabled detection of cellular metabolic activity. The 2040–2,300 cm^−1^ spectral region contains no intrinsic cellular signals. However, by providing deuterated substrates, active cells integrate environmental D into newly synthesized biomacromolecules, producing significant peaks from the conversion of C–H to C–D bonds. C–D bond intensity has become a universal indicator (Berry et al., 2015). This Raman-DIP strategy was applied in a P-limited culture system, where insoluble P was the sole source. Only phosphate-solubilizing cells can acquire P, maintaining metabolic activity. The study confirmed that the C–D ratio (CDR) was positively correlated with soluble P content and acid phosphatase activity (Li et al., 2019). This method converts phosphate solubilizing functions into recognizable metabolic signals, offering a powerful tool for quantifying the in-situ P solubilizing capacity of the soil microbiome. Also, beyond P-cycling organisms, SCRS has demonstrated extensive utility in characterizing diverse microbial phenotypes, such as those involved in carbon/nitrogen cycling and antibiotic resistance (Hatzenpichler et al., 2020; Pan et al., 2025).

In summary, SCRS employs two complementary strategies, D_2_O-labeled metabolic tracing and endogenous fingerprint imaging. These strategies overcome the challenge of phenotypic identification of key functional groups in the P cycle, providing a powerful *in situ* analytical tool for studying P metabolic flux at the micro-scale.

### 3.2 SCRS reveals the metabolic mechanisms of *in situ* PSMs and PAOs

SCRS not only identifies PSMs and PAOs through functional peaks in Raman spectra but also provides *in situ* metabolic insights at single-cell resolution through non-destructive, real-time chemical imaging. For example, using the “Raman-D_2_O” strategy in soil matrices, it was shown that under P limitation, PSMs enter metabolic dormancy to minimize wasteful energy consumption (Li et al., 2024). Under these conditions, their phosphate-solubilizing activity can only be activated and enhanced when exogenous organic carbon is supplied. This indicates that PSMs adopt a strategy characterized by “enhanced carbon metabolism in exchange for P accessibility”. In contrast, PAOs exhibit complex mechanisms for intracellular P chelation and allocation. SCRS was first applied to study EBPR-related metabolites (poly-P, PHA and glycogen) in PAOs, validating the anaerobic release and aerobic uptake metabolic model at the single-cell level (Majed et al., 2012). SCRS further revealed that under extreme starvation stress, PAOs preferentially consume glycogen for maintenance energy, only hydrolyzing poly-P once glycogen reserves are depleted (Bucci et al., 2012).

SCRS corrected previous models of P metabolism by revealing microbial phenotypic heterogeneity *in situ*. For example, the P-solubilizing activity of soil PSMs varied from 2% to 30% (Li et al., 2024). This suggests that in the same microenvironment, only a fraction of PSMs maintain high metabolic activity to dissolve P, possibly as a survival mechanism. This highlights the critical need to shift from abundance-based to activity-based analyses, focusing on functionally active microbial subsets. SCRS also reveals microbial metabolic diversity. Using FISH-Raman, it was confirmed that *Tetrasphaera,* a key PAO in wastewater treatment, lacks PHA (Singleton et al., 2022). SCRS studies indicate that microbial diversity in P metabolism is far more complex than anticipated and cannot be fully understood through population-level analyses.

### 3.3 Mining of highly active *in situ* PSMs and PAOs strains by SCRS

Besides precise non-culture phenotypic identification, SCRS, combined with microfluidic chips and optical tweezers, enables the precise manipulation and sorting of targeted single cells. The sorted cells can be further studied in two ways: (i) coupling with low-bias nucleic acid amplification to generate high-coverage genomic data linked to metabolic phenotypes, which is particularly advantageous for elucidating the genetic functions of difficult-to-culture or rare taxa (Xu et al., 2020; Wang et al., 2021; Zhang et al., 2023), and (ii) direct single-cell cultivation targeting *in situ* metabolic activity, which helps avoid competitive contamination and improves the success rate of isolating *in situ* functional microorganisms (Jing et al., 2022; Jing et al., 2025).

**Figure 1:**
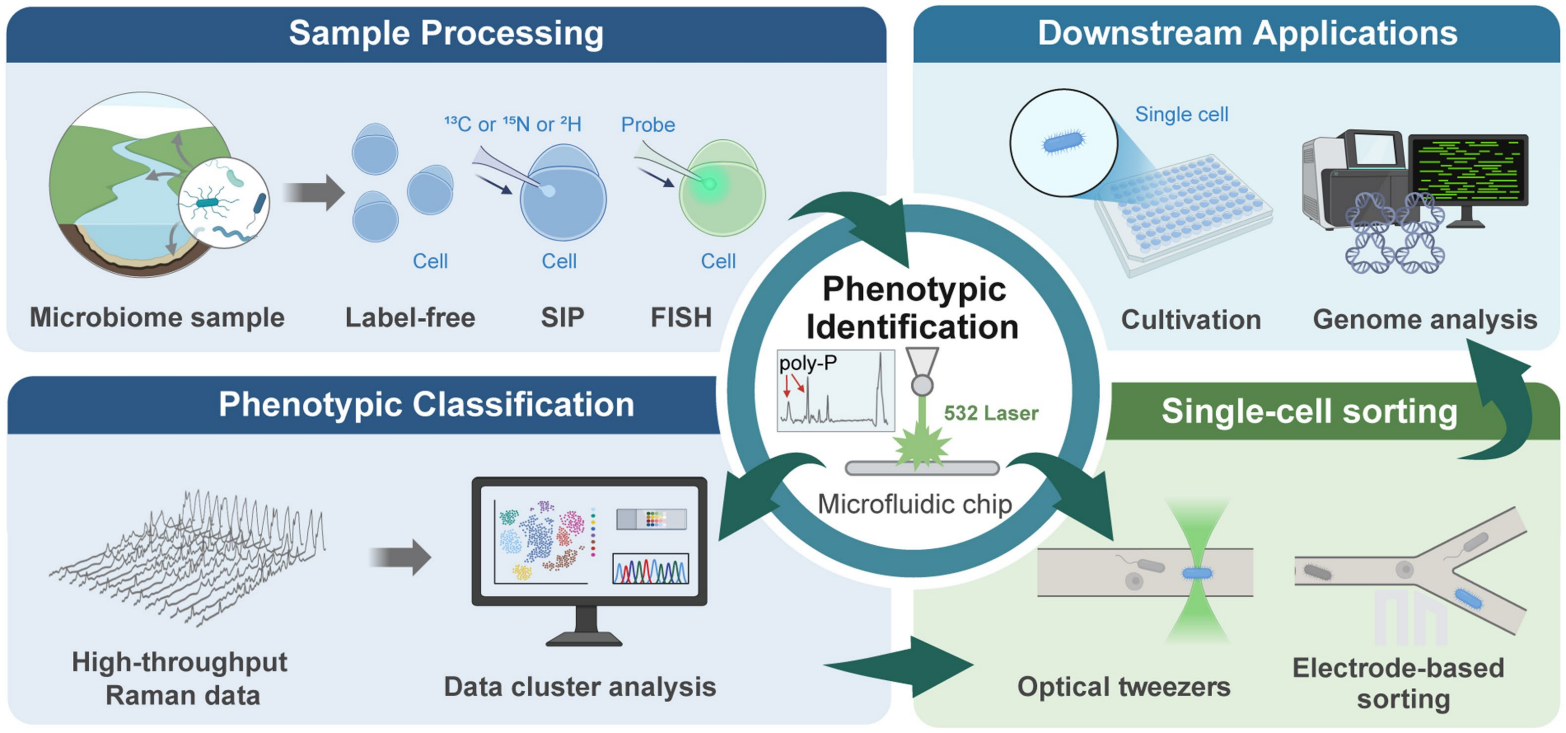
SCRS-based single-cell pipeline for P-cycle phenotyping and resource mining. (i) Sample processing: environmental microbiomes treated label-free or with SIP/FISH probes. (ii) Phenotypic classification: Raman spectra clustered to identify functional groups (e.g., PAOs). (iii) Single-cell sorting: optical tweezers or microfluidics isolate phenotype-targeted cells. (iv) Downstream applications: single-cell sequencing or targeted cultivation links phenotype to genotype and recovers strains.

This strategy has successfully isolated highly active PSMs from various habitats. For instance, using Laser-Induced Forward Transfer (LIFT) for single-cell sorting, cells with high phosphate-solubilizing activity were isolated. Subsequent 16S rRNA sequencing and metagenomic analysis identified previously overlooked low-abundance soil taxa, including *Bacillus marmarensis*, *Moraxella osloensis*, and *Stenotrophomonas maltophilia*, further revealing the genomic-level metabolic mechanisms of soil PSMs in the P cycle (Li et al., 2024). A “screen-first culture-second” strategy via single-cell Raman-activated sorting and cultivation (scRACS-culture) successfully isolated efficient PSMs such as *Comamonas* spp., *Acinetobacter* spp., *and Citrobacter* spp. from wastewater (Jing et al., 2022). Notably, PSMs activity under *in situ* conditions was 1–2 times higher than in pure culture, highlighting the significant value of this strategy in reflecting microbial *in situ* functions (Jing et al., 2022). Similarly, the scRACS-Culture workflow was used to isolate highly active PAOs, including *Acinetobacter* spp., *Micrococcus luteus*, and *Bacillus* spp. Notably, *Micrococcus luteus*, a novel PAO, showed ~14.8% of its poly-P accumulation in pure culture compared to *in situ* (Jing et al., 2025). This significant “phenotypic degradation” explains why it is often missed in traditional screenings. When applied to an anaerobic-anoxic-aerobic reactor treating municipal wastewater, this strain significantly improved P removal efficiency from 45% to 89%. These findings indicate that *in situ* metabolic activity-based screening can uncover key functional strains overlooked by traditional methods, thereby providing valuable resources for wastewater ecosystem engineering (Figure 1).

## 4 Application potential of SCRS in MRB strategy

The stable implementation of the MRB strategy relies on two key points. First, identifying and discovering highly efficient microbial resources that drive P cycle transformation into complex habitats, forming the biological foundation for sustainable P cycling. Second, constructing and regulating microbial communities where PSMs and PAOs act synergistically to optimize P resource transformation and recycling. Chapter 3 of this study highlights the advantages of SCRS in identifying efficient microbial resources and metabolic characterization. For community construction and regulation, SCRS can simultaneously quantify carbon (C) and P metabolites (e.g., PHA, glycogen, C–D, poly-P) at the single-cell level, offering unique insights into the perspective for deciphering this complex C–P interaction (Jing et al., 2021; Bi et al., 2025; Jing et al., 2025).

Research shows that high P-solubilizing activity in PSMs is linked to strong carbon assimilation and is regulated by environmental P levels (Li et al., 2024). In practical applications, precise control of the C/P ratio is essential. SCRS monitors the intracellular poly-P/PHA intensity ratio in PAOs as a regulatory indicator. If PHA accumulates while poly-P stagnates, it indicates a ‘C-rich, P-starved’ state, and the “Mobilization” effect of PSMs should be enhanced. If the C–D signal of PSMs declines, it indicates insufficient or unsuitable carbon sources, requiring timely replenishment and replacement. Due to the high specificity of PSMs and PAOs for carbon substrates (Zhao et al., 2022; Li et al., 2024), SCRS can aid in the targeted cultivation of functional microbial communities. By feeding different carbon sources and monitoring C-D signal responses, specific carbon sources that activate high-activity rare PSMs in soil can be identified, avoiding waste from indiscriminate carbon addition. Furthermore, the local anaerobic microenvironment that may be generated during PSMs activity provides ideal conditions for the anaerobic P release of PAOs. The P solubilization by PSMs and P release by PAOs form a dual P supply. PAOs are diverse; for example, fermentative PAOs match specific PSMs, and the small molecular carbon sources produced after fermentation by these PAOs will further activate the P-solubilizing activity of PSMs, thereby achieving maximum P activation efficiency with minimal carbon input (Wang et al., 2023a).

During the “Retention and Buffering” stage, the goal is to maintain a dynamic “biological P buffer pool” through PAOs, which sequester soluble phosphates as intracellular poly-P, thereby mitigating abiotic fixation or leaching losses. These poly-P reserves act as slow-release reservoirs. SCRS revealed a high abundance of PAOs in the rhizosphere (up to 30%), and interactions between *Ca. accumulibacter* phosphatis and Plant Growth-Promoting Rhizobacteria (PGPR) (He et al., 2025). Studies show that poly-P-rich fertilizers promote crop growth more effectively than traditional fertilizers, highlighting PAOs’ underexplored role in agriculture. Similarly, in wastewater treatment, the “Mobilization” effect of PSMs enables PAOs recover total P, thereby indirectly enhancing overall P removal. SCRS also confirmed the presence of numerous PSMs in wastewater (Jing et al., 2022), but their contribution to P removal and their synergistic mechanisms remain underexplored. In summary, although the MRB strategy needs further validation, the high-resolution metabolic fingerprinting, particularly for PAOs and PSMs’ key metabolites (e.g., poly-P, Glycogen, and PHA), provides valuable insights for precise regulation of P resource recycling.

## 5 Conclusions and perspectives

Given the global shortage of P resources, rising fertilizer demand, and the eutrophication crisis caused by P-containing wastewater, this review proposes the MRB strategy as a key solution to balance P supply and demand. The strategy centers on the synergistic interaction between PSMs and PAOs. We highlight that SCRS has significant advantages for *in situ* phenotypic identification of PSMs and PAOs, metabolic analysis, and strain resource mining. By enabling quantitative analysis of key metabolic substrates, SCRS is expected to guide the precise regulation and implementation of the MRB strategy (Jing et al., 2021; Bi et al., 2025; Jing et al., 2025). Future work should integrate scRACS-culture to isolate highly efficient *in situ* PSMs and PAOs from diverse habitats, providing core strains for MRB. These strains can be further used to construct composite microbial communities, and their competitive advantage is expected to be key to achieving industrial application. It should also establish Raman-based real-time monitoring platforms (Wang et al., 2020; Zhang et al., 2024), such as monitoring the biological P removal efficiency in wastewater and the activity status of soil PSMs, to enable precise early warning and regulation based on microbial physiological states before system failure.
